# Trends in life science grid: from computing grid to knowledge grid

**DOI:** 10.1186/1471-2105-7-S5-S10

**Published:** 2006-12-18

**Authors:** Akihiko Konagaya

**Affiliations:** 1 Genomic Sciences Center, RIKEN, Yokohama, Japan

## Abstract

**Background:**

Grid computing has great potential to become a standard cyberinfrastructure for life sciences which often require high-performance computing and large data handling which exceeds the computing capacity of a single institution.

**Results:**

This survey reviews the latest grid technologies from the viewpoints of computing grid, data grid and knowledge grid. Computing grid technologies have been matured enough to solve high-throughput real-world life scientific problems. Data grid technologies are strong candidates for realizing "resourceome" for bioinformatics. Knowledge grids should be designed not only from sharing explicit knowledge on computers but also from community formulation for sharing tacit knowledge among a community.

**Conclusion:**

Extending the concept of grid from computing grid to knowledge grid, it is possible to make use of a grid as not only sharable computing resources, but also as time and place in which people work together, create knowledge, and share knowledge and experiences in a community.

## Introduction

Bioinformatics applications often require high-performance computing and large data handling which exceeds the computing capacity of a single institution [1]. Sharing of unpublished data is also important in promoting collaborative research among institutions, as well as sharing of public databases, bioinformatics tools and web services [2-7]. Biological knowledge, such as ontology and meta data, also plays an important role in analysis of experimental data and integrating genome-wide OMICS data including genome, transcriptome, proteome, and other types of data [8,9]. Grid computing is a promising information technology which meets the above requirements, and has great potential to become a standard cyberinfrastructure for life sciences [10,11]. However, many features of it remain to be improved in terms of availability, performance and security, to name a few.

This paper reviews the latest grid technologies for life sciences mainly from papers published in the proceedings of international conferences: LS-GRID2004 [12], LSGRID2005 [13], LSGRID2006 [14], CCGRID2006 [15] and NETTAB2006 [16].

The grid technologies can be classified into three categories from the viewpoint of application development: computing grids, data grids, and knowledge grids. Although the grid is general enough to execute any type of life science application, the above classification is helpful for understanding the pros and cons of grid technologies when they are used for real life science applications.

The organization of this paper is as follows. The section, "Computing grid" introduces computing grid technologies focusing on virtual screening and large-scale sequence matching from the viewpoint of high-throughput computing. The next section, "Data grid" focuses on data grid technologies from the viewpoints of service integration, workflow and security when assuming open grid service architecture (OGSA). The "Knowledge grid" section discusses the requirements of knowledge grid technologies when using a grid as a cyberinfrastructure for knowledge creation based on the Nonaka knowledge spiral between explicit knowledge and tacit knowledge. Finally, a summary of the current status and future perspectives of life science grid technologies is presented.

## Computing grid

Bioinformatics applications often have to deal with thousands of relatively small independent tasks, each of which costs at most seconds or minutes for computation. This type of computation is referred to as "high throughput computing" and is distinguished from "high performance computing", which aims at short turnaround time on large scale computing using parallel processing techniques and special purpose computers [17,18].

Although grid computing aims at parallel and distributed computing, like cluster computing, the two differ in network latency and robustness. Network latency among institutions is far longer than that in a system area network in clusters even if network throughput performance is the same, for example, a giga-bit per second. In addition, the frequency of remote task failures is much higher in grid computing than in cluster computing due to the overhead of remote task invocation and the heterogeneity of computation pools. Therefore, handling of unexpected node termination and network problems is mandatory in grid computing, especially for lengthy execution jobs which take weeks and months of total time. There are two types of high-throughput computing in life sciences: numerical processing, typified by virtual screening, and symbolic processing, typified by sequence matching.

### High throughput numerical processing

High throughput numerical processing has become popular in bioinformatics due to the emergence of systems biology, which aims at modeling of biological dynamics in molecules, cells, organs and individuals. Huge computational power is necessary for the simulation of molecular folding, molecular docking, and spatiotemporal molecular interaction, and for the kinetic parameter estimation of metabolic pathways and signal transduction pathways, and so on. Problem decomposition techniques such as parameter sweep and stochastic modeling are often used to obtain a set of independent tasks in life science applications.

One of the best examples of life science high-throughput computing is the WISDOM high-throughput docking project in the Enabling Grids for E-sciencE (EGEE) project. It achieved over 46 million docking simulations, using 1700 computers distributed in 15 countries in about 6 weeks. The equivalent of 80 years on a single machine was used to find new inhibitors for a family of proteins produced by *Plasmodium falciparum *from 11 July 2005 to 19 August 2005 [19].

DIANE is an enhanced version of WISDOM with a light-weight framework. It was used to search for potential drugs for the predicted variants of the *avian flu *virus (H5N1), and produced two millions docking complexes with a size of 600 gigabytes using 2000 grid worker nodes distributed in 17 countries [20].

The above virtual screening projects revealed the limitations and bottlenecks of the current EGEE infrastructure. Overall grid efficiency was reported to be about 50 percent, on average. Server license failure, workload management failure and site failure were major sources of failures with rates of 23, 10 and 9 percent, respectively [21]. This means that much remains to be accomplished in grid middle-ware in improving availability and performance in solving real-life science problems.

Another example of high-throughput computing in bioinformatics is parameter estimation of ordinary differential equations for the mathematical modeling of metabolic pathways and signal transduction pathways [22]. Genetic algorithms are often used for estimating optimal parameter fitting to biological experimental results [23-25]. Genetic algorithms exhibit high degrees of parallelism, since they require multiple trials with various initial conditions as well as fitting function evaluation for each individual on each generation.

"Parameter Mining" is an alternative approach to genetic algorithms for the parameter estimation of mathematical models [26]. It uses two-dimensional geometrical patterns representing parameter-parameter dependencies (PPD) in differential equations, obtained by calculating moment parameters, such as area under the curve (AUC), mean residence time (MRT), and variance of residence time (VRT). Each two-dimensional pattern requires 25*21 measurement points to cover (10 to 6)*(10 to 5) parameter ranges, and 370 Gigabytes and 71 single cpu days are required for calculation of 256 geometrical patterns with 2,150,400 simulation in total. This CPU and data-intensive approach enables more precise mapping of biological experimental data on appropriate locations in geometrical patterns with a bird's eye view.

### High throughput symbolic processing

Sequence analysis, such as homology searches, genome comparisons and genome-wide analyses, are typical examples of time-consuming high-throughput symbolic processing applications in bioinformatics. Although the human genome sequence project has been concluded, there is still strong demand for high-performance sequence analysis due to the emergence of metagenomic projects and human resequencing projects as well as genome sequencing projects on mammalian and other species [27]. Sequencing data are expected to increase more rapidly as high-throughput DNA sequencing technologies become popular and economical.

Unlike numerical processing, bioinformatics symbolic processing often requires large databases such as DNA and protein sequence databases. Sharing and updating of biological databases on the grid are of key importance in high-throughput symbolic processing such as homology searches, genome comparison and genome-wide scan analyses.

#### Sharing and updating of biological databases

Sharing and updating of biological databases has become more and more difficult and intractable due to the rapid increase in DNA and genome sequence data. Rapid progress of DNA chip technologies also contributes to the expansion of gene expression databases and SNP databases. Automatic updating of databases is necessary to decrease the database maintenance costs, especially when the number of replicas becomes large in grid [28]. In the deployment of genome databases on worker nodes, duplicated database copying, disk overflow, unexpected shutdown, version management, and file checksum integrity verification are all concerns, as well as parallel and pipelined mechanisms for high-throughput data transfer [29].

EGEE also provides a general framework for sharing replicas of biological databases represented by logical filenames (LFNs) using a replica manager system (RMS). The framework enables execution of bioinformatics applications on computing elements with randomly replicated LNFs on the storage elements of several grid nodes shared by more than 30,000 CPUs in total [30].

The Genome Analysis and Database Update system (GADU) provides an automated, scalable, high-throughput computational workflow engine that executes bioinformatics tools (BLAST, BLOCKS, PFam, Chisel and InterPro) with public databases (NCBI RefSeq, PIR, InterPro and KEGG) on multiple Grids of different architectures and environment, a collective member of more than 18,000 CPUs contributed by more than 60 institutions [31].

#### Homology search

BLAST is a typical example of high-throughput symbolic processing in homology searches. Many GRID BLAST implementations have been developed and reported [30-35]. The characteristics of Grid Blast are summarized as follows: (1) prestaging of sequence databases to minimize the runtime overhead of transferal of large sequence databases, which often reach several Gigabytes in size, (2) databases update which keeps data consistency on the data-grid, (3) dynamic load balancing of query sequences to avoid unexpected slow responses, especially when dealing with thousands of query sequences in heterogeneous computation pools including PC-clusters and desktop computers, and (4) assembling of the results from distributed jobs.

#### Genome comparison

Genome comparison is one of the most promising life science applications for grid computing. "The computation will be left behind a tidal wave of genomic data, unless an expandable and flexible large scale computing facility is established" described Sugawara, when investigating horizontal gene transfer among 354,606 ORFs extracted from more than 100 microbial genomes using 229 CPUs located in five institutions in 2003 [36]. It should be noted the number of pair-wise sequence comparison increases in proportion to the square of the number of genome sequences. Grid is one of feasible information technologies that can provide huge computation power necessary for this purpose.

#### Genome-wide scan analysis

Genome-wide scan analysis becomes more and more important but time-consuming in nature. Recent disccovery of RNA world reveals the importance of finding highly conserved regions in genome sequences for non-coding genes and microRNA binding regions as well as coding-genes and binding factor regions. SNP-based population genetics and copy number analysis on genome sequence variations are also important applications for a life science grid in near future. Gridification of sequence analysis tools are urgent issues to deal with ever–expanding genome sequences [37,38].

## Data grid

"We suggest that the full set of bioinformatics resources–the *resourceome*–should be explicitly characterized and organized." noted Russ Altman in his article [8]. Resourceome requires a uniform interface in which all the bioinformatics databases and application tools can be accessed through web services and workflow systems in a secure fashion. Ontology and/or meta data are also required to integrate the bioinformatics services. Data Grids based on Open Grid Service Architecture (OGSA) are beginning to satisfy the above requirements, and will be applicable to practical applications including pharmacogenomics and clinical-trials in the near future.

### Integration of bioinformatics services

OGSA provides a general framework for sharing of resources among institutions over firewalls based on the Web Service Resource Framework (WSRF). It enables execution of bioinformatics applications and workflows with remote resources through web services in secure fashion. Metadata and ontology play an important role to fill the semantic gap of heterogeneous databases as follows.

The Japanese BioGrid project designed application metadata and data service metadata to fill the semantic gap among gene-protein databases, interaction databases and compound databases necessary for drug-design using GT3 and OGSA-DAI for the implementation of a heterogeneous database federation [39]. The @neurIST project developed a service-oriented grid infrastructure to integrate public databases, hospital information, private databases, modeling and simulation using Web Service Level Agreements (WSLA) for QoS-enabled computer service [40].

The Sealife project aimed at context-based information integration on a semantic web/grid browser which automatically links a host of web servers and Web/Grid services to the Web content being visiting. Text mining and concept mapping techniques were used for bridging the gap between the free text on the current web and the ontology-based mark-up for the semantic web and the grid services [41].

#### Bioinformatics workflow

Bioinformatics workflow tools are necessary for end-users to make use bioinformatics web/grid services. Taverna is one such example which provides a workflow language and graphical user interface to facilitate the easy building, running and editing of workflows allowing the integration of resources that are published as Web services [42]. However, the quest for resources becomes a very demanding and time-consuming activity, so that a dynamic semantic indexing system of bioinformatics services becomes essential [43]. Searching functionally similar bioinformatics workflows is also important for the recyclable use of bioinformatics workflows [44]. In addition, automatic generation of bioinformatics is possible if bioinformatics ontology that defines input-output data specification and functional specification is established [45].

A workflow management system is also helpful for deploying grid applications because it enables to encapsulate architectural differences of heterogeneous grid resources from application users [46-48]. Agents society is another approach to integrate insilico experiments, resource discovery and biological system simulation [49].

#### Secure data access

Many bioinformatics databases are public and freely available, but it is often the case that access to the data needs to be strictly controlled in distributed collaborative research. A secure framework is needed to access clinical data that exists across regional, national and international boundaries for clinical trials and unbiased evaluations of their outcome [50]. Although Public Key Infrastructures (PKI) is the predominant method for enforcing authentication in a grid community, the Virtual Organization for Trials and Epidemiological Studies (VOTES) project adopted the Internet2 Shibboleth technology to allow a "single sign-on" authentication step between the grid/data servers and the local database resources [35,50,51].

## Knowledge grid

Michael Polanyi, a 20th-century philosopher, commented in his book, *The Tacit Dimension*, that "we should start from the fact that we can know more than we can tell". This means that knowledge which we can represent on computers is just a part of knowledge which we can create, transfer and share among a community.

The Grid can be considered as a kind of "Ba", a Japanese philosophical concept, that conceptualises time and place where people work together and create knowledge [9]. This "ba" can be designed not only for sharing explicit knowledge but also for sharing tacit knowledge among communities and/or virtual organizations [52].

According to the Nonaka knowledge spiral theory [53], knowledge creation requires a cyclic process of knowledge conversion between tacit knowledge and explicit knowledge; (1) Socialization (tacit knowledge to tacit knowledge), (2) Externalization (tacit knowledge to explicit knowledge) (3) Combination (explicit knowledge to explicit knowledge) and (4) Internalization (explicit knowledge to tacit knowledge). This has significant insights into what it will take to support the realisation of the Grid amongst our scientific community. This framework gives a meta-philosophical approach to rationalise the current Grid phenomemon.

### Socialization

Socialization is the first step in formulating a community. Grid portals are helpful for attracting those who are interested in some specific field. However, the role of a portal will be limited if it does not allow formulation of user-defined communities. Knowledge grids should provide social communication system-like facilities in which any participant can formulate a new community and can recruit other participants. Face-to-face meeting or off-site meeting will be also helpful in promoting mutual understanding in a community.

### Externalization

Externalization is the essence of knowledge creation. It is not too much to say that all research activities are a kind of externalization involving publication of research papers as a final result. In this sense, knowledge grid should provide facilities for participants to publish their knowledge in a community. Web-based dynamic contents are one of the promising ways of publication of knowledge [54].

### Combination

Combination expands knowledge by the sharing of explicit knowledge in a community. Synergy effects can be expected if participants bring together their own knowledge. Grid portals [55-57] and application-oriented grids [58-61] play an essential role in this process.

### Internalization

Internalization is a process of acquiring tacit knowledge by experience. In order to make use of a grid for real world life science problems, a global bioinformatics environment, that is, a problem solving layer for bioinformatics must be developed on a grid. Gridfication of public databases and bioinformatics tools are necessary conditions but not sufficient for this. The bioinformatics environment should provide secure facilities to deal with unpublished data and customization facilities to develop one's own bioinformatics environment coordinated with global bioinformatics environment.

## Conclusion

Computing grid technologies have been matured enough to solve high-throughput real-world life scientific problems like virtual screening of docking simulation. Scalable distributed storage management systems are also necessary to deal with high-throughput sequence analysis on ever-increasing DNA sequence data.

Data grid technologies are strong candidate for realizing *resourceome *for bioinformatics. OGSA and workflow management system enable to develop a global bioinformatics environment in which any biological databases and bioinformatics tools can be access through grid services. Ontology and common data-exchange formats are keys to establish interoperability among bioinformatics grid services.

Knowledge grid should be designed not only from sharing explicit knowledge on computers but also from community formulation for sharing tacit knowledge among a community. Then, we can extend the concept of grid as a *ba*, that is, time and place in which people work together, create knowledge, and share knowledge and experiences in a community.
